# Natural Course of Fulminant Hepatic Failure: The Scenario in Bangladesh and the Differences from the West

**DOI:** 10.4103/1319-3767.56094

**Published:** 2009-10

**Authors:** Shahinul Alam, Golam Azam, Golam Mustafa, Abul Kalam Azad, Izazul Haque, Shakil Gani, Nooruddin Ahmad, Khorshed Alam, Mobin Khan

**Affiliations:** Department of Hepatology, Bangabandhu Sheikh Mujib Medical University, Shahbag, Dhaka, Bangladesh; 1 Department of Immunology, Bangladesh Institute of Research and Rehabilitation in Diabetes, Endocrine and Metabolic Disorders, Shahbag, Dhaka, Bangladesh; 2 Department of Medicine, Bangabandhu Sheikh Mujib Medical University, Shahbag, Dhaka, Bangladesh

**Keywords:** Fulminant hepatic failure, hepatitis E virus, Bangladesh, acute hepatitis E, transplantation

## Abstract

**Background/Aim:**

Fulminant hepatic failure (FHF) is a devastating complication of acute viral hepatitis, leading to death in most cases. The etiology and predictors of outcome differ according to the geographical region. This study was conducted with the aim of evaluating the etiology, complications, and outcome of FHF in Bangladesh.

**Patients and Methods:**

In this prospective study, we included 67 consecutive cases of FHF presenting to the Department of Hepatology, Bangabandhu Sheikh Mujib Medical University, Dhaka, between November 2003 and May 2008. Thirty-nine of the patients were male and 28 were female. Data was analyzed using SPSS, version 13.0.

**Results:**

The mean age of the subjects was 31.9 ± 11.7 years. Hepatitis E virus (HEV) was the commonest etiological factor for FHF (50 cases, 74.6%); of the 50 cases with HEV infection, 43 (64.2%) were not coinfected with any other virus, four cases were Hepatitis B virus (HBV) carriers, and three had coinfection with hepatitis A virus (HAV). HBV was the cause of FHF in nine (13.4%) patients. HCV, paracetamol, and alcohol were not responsible for any of the cases. Most of the patients (57 patients, 85%) developed FHF within 2 weeks of the onset of jaundice. Of the 67 patients, 49 (73.1%) died. Cerebral edema was the single most common cause of death (48 patients, 71.6%). Other complications were renal failure (23 patients, 34.3%), sepsis (15 patients, 22.4%), electrolyte imbalance (12 patients 17.9%), and bleeding tendency (7 patients, 10.4%). Occurrence of cerebral edema, longer prothrombin time, higher grade of encephalopathy, and longer jaundice-to-encephalopathy interval had significant negative influence on outcome.

**Conclusions:**

The etiology of FHF in Bangladesh is different from that in the West. Prolongation of prothrombin time and occurrence of cerebral edema are predictors of the worst prognosis.

In 1970, Trey and Davidson defined fulminant hepatic failure (FHF) as the occurrence of encephalopathy within 8 weeks of the onset of acute hepatitic illness in an individual without preexisting liver disease.[1] It is the most fatal complication of acute hepatitic illness resulting from various causes.[2,3] Hepatitis B infection still remains the most important cause of FHF in Greek patients.[4] The most common cause of FHF in Western countries is acetaminophen overdose. In developing countries, on the other hand, hepatotropic viruses- hepatitis A or hepatitis B in the Far East[5] and hepatitis E in India and Bangladesh[6–8]-seem to be the dominant cause.

The mortality rate of FHF is 80% without liver transplantation; this decreases to 30% when the facility of liver transplantation and artificial liver support system is available. The natural history is short and there are no chronic sequelae in survivors.[4,9] So the quality of life is far better in survivors who have not undergone liver transplantation. Most of the data on FHF have been reported from USA,[10] UK,[11] the Far East,[2,12] and France.[13] The etiology and predictors of outcome in Western populations differ from that in developing countries.

Bangladesh, with a total population of about 150 million, is a densely populated country. The complete picture with regard to FHF in Bangladesh has not been previously reported.This study was designed to address the etiology, complications, outcome, and predictors of outcome of FHF in Bangladesh.

## PATIENTS AND METHODS

### Patients

In this prospective study, we included 67 patients of FHF who were admitted to the Department of Hepatology of the Bangabandhu Sheikh Mujib Medical University, Dhaka, Bangladesh, during the period from November 2003 to May 2008. The patients were referred to our center from peripheral primary and secondary care hospitals in all parts of Bangladesh.

### Inclusion criteria

The diagnosis of FHF was based on the occurrence of hepatic encephalopathy within 8 weeks of onset of jaundice in patients with no previous liver disease and the presence of coagulopathy as proved by a prothrombin time > 15 s or international normalized ratio (INR) > 1.5].[14]

### Exclusion criteria

Patients in whom the history was not available or the diagnosis was not confirmed and those patients with preexisting liver disease were excluded from the study.

### Working definitions

#### Jaundice-to-encephalopathy interval

The interval (in days) from the detection of icterus to the onset of hepatic encephalopathy.

#### Survival time

The time from onset of encephalopathy till complete recovery from encephalopathy or, alternatively, till death.

#### Cerebral edema

Cerebral edema was defined by the presence of spontaneous decerebrate posturing or the presence of any two of the following four factors, i.e., hypertension (supine blood pressure ≥ 150/90 mm Hg), bradycardia, pupillary changes, and presence of neurogenic hyperventilation.[2]

#### Renal failure

Renal failure was defined as a progressive increase of serum creatinine concentration (>2 mg/dl), with or without oliguria, as a result of functional renal failure, acute tubular necrosis, or the hepatorenal syndrome.

#### Coagulopathy

Coagulopathy was diagnosed on the basis of a prolonged prothrombin time (patient's value 4 seconds more than that of control), with or without bleeding manifestations.

#### Infection

Infection was diagnosed if the body temperature was >101°F or <98°F and there was neutrophilic leukocytosis or at least one of the following: Presence of pneumonia or identification of pathogenic microorganism in cultures of blood, urine, or other body fluids.

All initial assessments were by residents of hepatology; the findings were confirmed by senior hepatologists (professors or associate professors).

### Treatment protocol

All patients were evaluated with a detailed history and clinical examination at admission; the clinical examination was repeated every 1-2 h thereafter. All patients were given standard supportive treatment and admitted to the intensive care unit for careful monitoring. Treatment included maintenance of fluid and electrolyte balance, adequate nutrition, appropriate antibiotics, lactulose, an injectable proton pump inhibitor, parenteral mannitol, and ventilatory support. Facilities for artificial liver support and liver transplantation are not currently available in Bangladesh.

### Investigations

The etiological diagnosis was made on the basis of laboratory investigations and clinical information obtained at admission. Patients were screened for serological markers of hepatitis A, B, C, and E viral infection, including immunoglobulin (Ig) M antibodies against HAV and HBV, and for autoantibodies (antinuclear, anti-liver/kidney microsome, and anti-smooth muscle autoantibodies). IgM anti-HAV, HBsAg, IgM anti- HBc, anti-HCV, anti-HEV IgM were tested for in patients' sera using ELISA. Wilson's disease was evaluated by the standard criteria of serum ceruloplasmin levels, copper in serum and urine, presence of Kayser-Fleischer ring, and family history. The diagnosis of ‘unknown etiology’ was considered after complete workup had excluded all known etiologies.

### Statistical methods

Data entry and analysis were carried out using SPSS 13.0 (SPSS, Chicago, IL, USA). Inter-group comparisons for categorical variables were done using the chi square test with Fisher's exact test and those for quantitative variables were compared by the independent ‘*t*’ test. The prognostic factors for outcome were determined with logistic regression analysis. A *P* value less than 0.05 was considered significant.

## RESULTS

### General characteristics

We had a total of 67 cases of FHF in our study, of whom 39 (58.2%) were males. The age range was 14-70 years, with 53 patients (79.1%) being ≤40 years of age. The highest bilirubin level, ALT level, and prothrombin time over control (patient value - control value) was 16.9 ± 10.6mg / dl, 1202.9 ± 048.9 U/l, and 42.4 ± 21.3 s, respectively. During the treatment period highest grade of encephalopathy of the individual patients were grade I, II, III and IV were 2 (3%), 12 (17.9%), 6 (9.0%) and 47 (70.1%), respectively. Out of the 28 female patients in our sample, 10 were pregnant; five of them were in the third trimester, two in the second trimester, and three in the first trimester of pregnancy. All the patients in this series developed FHF within 1 month of detection of jaundice and most of them (57 patients, 85%) developed FHF within 2 weeks of onset of jaundice [Table 1].

**Table 1:** Characteristics of the study population

Character	(%)
Total population	67
Age in years (mean ± SD)	31.9 ± 1.7
Gender (male/female)	39/28
Highest bilirubin level in mg/dl (mean ± SD)	16.9 ± 10.6
ALT level in U/l (mean ± SD)	1202.9 ± 1048.9
Prothrombin time over control in seconds (patient value - control value) (mean ± SD)	42.4 ± 21.3
Jaundice-to-encephalopathy interval in days (mean ± SD)	9.3 ± 6.8
Pregnancy	10 (14.9)
Etiology	
HEV	43 (64.2)
HBV	9 (13.4)
HEV on HBV carrier	4 (6.0)
Non A to E	4 (6.0)
HAV and HEV	3 (4.5)
HAV	2 (3.0)
Drug	2 (3.0)
Complications	
Cerebral edema	48 (71.6)
Renal failure	23 (34.3)
Infection	15 (22.4)
Electrolyte imbalance	12 (17.9)
Bleeding	7 (10.4)
Outcome	
Death	49 (73.1)

HEV: Hepatitis E virus; HBV: Hepatitis B virus; HAV: Hepatitis A virus

### Etiology of fulminant hepatic failure

HEV, which was responsible for the development of FHF in 50 patients (74.6%), was the commonest culprit in this study; of the 50 patients with HEV infection, 43 (64.2%) were not coinfected with any other virus, four were hepatitis B carriers, and three had coinfection with HAV. HBV, which was responsible for 9 (13.4%) cases of FHF, was the second commonest cause of hepatic failure. No cause could be identified in 4 (6.0%) cases. In this study, the medications that were implicated in the etiology of FHF were antitubercular drugs and herbal medicines. HCV, paracetamol, and alcohol were not responsible for any case of FHF in this study.

### Complications and outcome

Among the 67 patients in this series, 49 (73.1%) had a fatal outcome death and only 18 (26.9%) survived. Death occurred within 97.6 ± 74.5 h of the development of encephalopathy. Cerebral edema, the single most common cause of death, occurred in 48 (71.6%) cases. Other complications were renal failure 23 (34.3%), sepsis 15 (22.4%), electrolyte imbalance 12 (17.9%), and bleeding manifestation 7 (10.4%). Etiology, sex, age, and pregnancy had no statistically significant influence on outcome. Development of cerebral edema, longer prothrombin time, higher grade of encephalopathy, longer jaundice-to-encephalopathy interval, and lower ALT level had significant negative influence on outcome [Table 2]. Multivariate logistic regression analysis showed that ALT level and jaundice-to-encephalopathy interval had no significant effect on outcome.

**Table 2:** Comparative characteristics of survivors and nonsurvivors

Character	Survivors 18 (26.9%)	Nonsurvivors 49 (73.1%)	P*
Age (years)	31.9 ± 13.7	31.9 ± 11.1	1.0
Sex (male/female)	2/6	27/22	0.58
Etiology			
HEV+	15	28	0.08
HBV++	1	8	0.43
Non A-E	1	3	1.0
Encephalopathy			
Grade I-II	14	0	0.000
Grade III-IV	4	49	
Prothrombin time over control in seconds (patient value - control value)	18.6 ± 6.4	51.2 ± 17.8	0.000
Serum bilirubin (mg/dl)	18.8 ± 14.0	16.3 ± 9.1	0.39
ALT (U/l)	1638.6 ± 1138.4	1036.1 ± 974.1	0.04
Jaundic-to-encephalopathy interval (days)	6.9 ± 3.7	10.1 ± 7.5	0.02
Cerebral edema	2	46	0.000
Pregnancy	4	6	0.14

* *P* value by Chi square with Fisher's exact test and independent *t*-test;

+ HEV: Hepatitis E virus;

++ HBV: Hepatitis B virus

## DISCUSSION

The etiology of FHF differs according to the geographical region. In our series, HEV hepatitis is the commonest cause of FHF. Although paracetamol toxicity is the leading cause of FHF in Western countries, being responsible for 39% of the cases in USA,[13] 73% in UK,[15] and 70% in Denmark,[16] FHF in developing countries is predominantly due to the various hepatitis viruses. All of the previous reports from the Indian subcontinent have identified infection with hepatitis viruses as the etiology in 95-100% of patients with FHF.[2,17–21] Of these, HEV is the most frequently implicated agent in the Indian population. Our findings are consistent with these reports as well as with a previous report from Bangladesh.[8] In industrialized countries, sporadic cases of acute hepatitis E occurs in individuals who have a history of travel to endemic areas. However, recent evidence suggests that such sporadic cases can occur in individuals who have no evidence of exposure to HEV strains from countries where the infection is endemic.[22] Evidence is accumulating that HEV infection could occur in developed countries via blood transfusion or as a zoonosis.[23,24]

HBV is responsible for a significant number of FHF cases in our study. HBV accounts for FHF in 27.6% of the cases in India, 20% in the USA, 2% in the UK, and 52.5% in Greece.[2,4,15] Paracetamol toxicity is the commonest cause of drug-induced FHF in developed countries but there was no such case in our study population; instead, antitubercular drugs and herbal medicines were causes of FHF in our sample. This is similar to the findings of other studies in this region but differs from the reports from developed countries.[15,17–21]

Earlier studies from Greece and some Western communities have reported that females are at an increased risk for developing FHF.[4] In our series, however, males were predominantly affected, a finding consistent with previous reports from Bangladesh and the Indian subcontinent.[7,8,18–20] This difference may be due to the fact that paracetamol self-poisoning, is the commonest cause of FHF in Western societies.

Eighteen patients in our series survived, giving an overall survival rate of 26.9%. This is far below the rates reported in Western studies[4] but very similar to the reports from this region.[8,12] As liver transplantation facilities and artificial liver support systems are not available in our setting, FHF follows its natural course, with supportive and intensive care management being the best that can be offered to patients.

Presence of cerebral edema, longer prothrombin time, and higher grade of encephalopathy had significant negative influence on survival. Age, sex, etiology, rapidity of onset of encephalopathy, highest bilirubin level, and pregnancy did not affect the outcome. Studies from the West have reported that the etiology of FHF and longer jaundice- to- encephalopathy interval have a negative influence on survival.[3,14,25–27] Our findings are not concordant with those studies but are similar to findings from other studies from this region.[7,8,17,18] Although pregnancy is recognized to adversely influence survival, in our series it did not affect the outcome, which is in accordance with a study by Acharya *et al*.[2] This may be because we had only a small number (10) of pregnant patients in our sample and 50% of them (five patients) were in the first and second trimester of pregnancy.

A major limitation of our study is the small sample size. Only two patients were found to have drug-induced hepatic failure.

## CONCLUSION

The etiology and outcome of FHF in Bangladesh differ from that in Western countries. The factors influencing outcome are also different from that in developed countries. Immediate measures should be taken to establish transplantation facilities in Bangladesh to reduce the high mortality rates in FHF. Further studies focusing on the influence of pregnancy on FHF is warranted.
